# Location probability learning in 3-dimensional virtual search environments

**DOI:** 10.1186/s41235-021-00284-3

**Published:** 2021-03-24

**Authors:** Caitlin A. Sisk, Victoria Interrante, Yuhong V. Jiang

**Affiliations:** 1 Department of Psychology, University of Minnesota, 75 East River Road, Minneapolis, MN 55455 USA; 2 Department of Computer Science, University of Minnesota, Minneapolis, MN USA

## Abstract

When a visual search target frequently appears in one target-rich region of space, participants learn to search there first, resulting in faster reaction time when the target appears there than when it appears elsewhere. Most research on this location probability learning (LPL) effect uses 2-dimensional (2D) search environments that are distinct from real-world search contexts, and the few studies on LPL in 3-dimensional (3D) contexts include complex visual cues or foraging tasks and therefore may not tap into the same habit-like learning mechanism as 2D LPL. The present study aimed to establish a baseline evaluation of LPL in controlled 3D search environments using virtual reality. The use of a virtual 3D search environment allowed us to compare LPL for information within a participant’s initial field of view to LPL for information behind participants, outside of the initial field of view. Participants searched for a letter T on the ground among letter Ls in a large virtual space that was devoid of complex visual cues or landmarks. The T appeared in one target-rich quadrant of the floor space on half of the trials during the training phase. The target-rich quadrant appeared in front of half of the participants and behind the other half. LPL was considerably greater in the former condition than in the latter. This reveals an important constraint on LPL in real-world environments and indicates that consistent search patterns and consistent egocentric spatial coding are essential for this form of visual statistical learning in 3D environments.

## Background

Visual search in real-world environments is a complex task that requires efficient shifts of attention, recognition of complex visual stimuli, and frequent reorienting as one turns to search all regions of the 3-dimensional (3D) space. Previous research has identified several mechanisms that support visual search, including search history (Awh et al. 2012). Imagine yourself standing in the center of your living room, searching for your keys, glasses, or the TV remote. Presumably, you have searched for these objects in this space before, and that search history may inform your current search (Oliva et al. 2004). However, the vast majority of research on selection history effects thus far lack one key component of real-world search environments: their 3D nature, which results in the need for search both in front of and behind one’s initial field of view.^1^ As a result, the mechanisms by which selection history guides search in 3D navigable environments, where search involves turning one’s body and reorienting, rather than just oculomotor movements, remain unclear. The current study investigates the role of selection history in visual search in 3D environments using virtual reality.

One well-researched selection history effect in small-scale, 2-dimensional (2D) environments is *location probability learning* (LPL). When a search target disproportionately appears in one “target-rich” region of a computer monitor, participants harness that statistical regularity, becoming faster at finding targets when they appear in that region than when they appear elsewhere (Druker and Anderson 2010; Geng and Behrmann 2002; Jiang et al. 2013; Miller 1988). LPL can guide search independently of conscious awareness (Jiang et al. 2018), and the attentional bias that participants develop toward the target-rich region is lasting and robust: participants retain this attentional preference for an extended period of time after learning (Jiang et al. 2013), the preference persists in the face of added cognitive load (Won and Jiang 2015), and aging and serious neurological conditions do not interfere with the acquisition or expression of LPL (Sisk et al. 2018). These findings suggest that LPL is supported by repeated shifts of attention in space, resulting in a robust attentional bias or preference akin to a search habit (Jiang and Sisk 2018).

The robust nature of LPL suggests that the effect should extend to search in large-scale 3D environments. Indeed, two lines of previous research have observed evidence of LPL in either visual search in complex outdoor environments (Jiang et al. 2014; Won et al. 2015) or foraging in an indoor controlled environment (Smith et al. 2010). In Jiang et al. (2014), participants searched for a coin on the ground in an outdoor search space. Unbeknownst to participants, the coin more often appeared in one region of the ground. Participants developed LPL, exhibiting not only faster search time but also an increased tendency to turn their head in the direction of the high-probability region. Smith et al. (2010) created a visually sparse indoor 3D environment that consisted of an empty room surrounded by black curtains with colored lights embedded into the floor. All of the lights were initially the same color. Participants had to press switches next to the lights embedded in the floor until they found the light that changed colors when the switch was pressed. Because the target was not defined by visual characteristics until the participant actively interacted with it, the task involved foraging rather than visual search. As in 2D LPL studies, the target appeared in one region—either the left or the right half of the room—more often (80% of all trials) than the other region (20% of all trials). Participants located the target more quickly in the “rich” side of the room if they began each search trial from the same starting point. Thus, despite increased size and complexity of the search environment, when the target frequently appears in one region of space, participants appear to have the capacity to acquire LPL.

Although LPL has been observed in both 2D computer-based experiments and 3D search or foraging tasks, it is unclear whether the effects observed in 3D tasks were the result of the same attention learning mechanism underlying LPL in 2D search tasks. Real-world search experiments (e.g., Jiang et al. 2014) were conducted in natural environments that provided rich visual cues, such as landmarks, which could lead to high levels of explicit awareness about the target’s location probability. The floor-light foraging task of Smith et al. (2010) did not contain rich visual cues, but the task took many seconds to complete and entailed complex foraging strategies, yielding a pattern of navigation behavior that distinguished it from visually guided search (Pellicano et al. 2011; Smith et al. 2008). In fact, participants in both Jiang et al. (2014) and Smith et al. (2010) achieved high levels of explicit awareness, with the majority of them correctly identifying the target-rich region when probed. If the learning effect observed in these 3D tasks is driven by explicit awareness of where the target-rich region is, then LPL in these 3D tasks may reflect goal-driven attentional guidance, rather than implicitly guided search habits.

Because implicit selection history effects can function independently of goal-directed attention (Awh et al. 2012; Jiang 2018), previous studies in these visually rich real-world search environments or foraging tasks may not represent an extension of the LPL that researchers have observed in 2D tasks. To understand LPL in 3D search environments, where search involves the space behind one’s starting position, it is necessary to first characterize LPL in highly controlled 3D environments in the absence of rich visual cues or complex foraging strategies. Only then can we systematically evaluate the influence of various environmental features, such as landmarks, navigability, and spatial scale on selection history effects in searching for items outside of the initial field of view.

The present study therefore used virtual reality to create a 3D search environment with three primary aims. First, we aimed to establish LPL in 3D environments with sparse visual cues. By eliminating the visual cues and landmarks present in previous studies, and by using visual search, rather than a foraging task, the present study can investigate LPL in a visually sparse 3D environment. This provides a “baseline” for future research on selection history effects in such large-scale environments. Although removing discrete visual cues eliminates the possibility of associating landmarks with target probabilities, it does not preclude participants from acquiring explicit awareness of the target’s location probability through learning the room geometry. Therefore, the second aim of the present study was to determine whether LPL in visually sparse 3D environments is associated with high levels of explicit awareness.

Finally, we aimed to test whether LPL is equivalent, regardless of whether target-rich locations appear in front of someone or behind that person. Although humans are capable of representing space that is outside of the field of view, the computational demands of attending to space in front us are different from the demands of attending to space behind us. Two primary computational distinctions can be noted. First, visual search among stimuli appearing in front of a participant is guided by perceptual properties of the search items. The integration of such perceptual representation with top-down goals is a hallmark of theories of visual search (Itti and Koch 2001; Treisman 1988; Wolfe et al. 2015). In contrast, stimuli behind a participant cannot be seen, depriving participants of bottom-up sources of attentional guidance and preventing them from engaging in visually guided search. Instead, search for regions behind someone is initially guided only by internal representations, with perceptual features of the search stimuli becoming available only after participants have physically oriented toward that region. Previous studies showed that participants tend to rely on perception, rather than memory in search tasks (O’Regan 1992). For example, participants frequently re-fixate the same object to extract different features of that object in visuomotor tasks instead of relying on memory for the object (Ballard et al. 1995; Droll and Hayhoe 2007). When participants searched within the same environment but for different targets, search was not more efficient, despite the familiarity of the search environment (Oliva et al. 2004). This may again reflect a tendency to rely on perceptual cues, rather than memory for the locations of objects within a familiar environment. A possible result of this tendency may be that people attend to space in front of them differently than to space behind them due to the availability of perceptual cues only for the space in front of them. This could, in turn, influence their ability to learn from prior selection history within those regions.

Second, because search of space behind oneself depends on physically turning one’s head or body, learning of a target-rich region behind oneself may be more challenging than learning of a target-rich region in front of oneself. Consider first a case in which the target-rich region is in the front right corner of the room relative to the initial perspective of a participant. Since this region is in front of the participant, they do not need to turn their body and reorient to find the target. Although the initial field of view does not include the entire front two quadrants of space if the search space is divided into four quadrants, participants in virtual reality studies typically use only head movements—not torso or body movements—when searching within this region (Sidenmark and Gellersen 2020). This region is therefore consistently referenced as “front right” on each trial where the target appears in that part of the room. Now consider a case in which the target-rich region is in the back right corner of the room, relative to the initial perspective of a participant. To find the target, the participant has to turn and reorient. Yet the lack of visually guided features prevents participants from knowing which direction to turn—clockwise or counterclockwise. Because the participant may turn clockwise on some trials and counterclockwise on others, and because the participant may turn 90º on some trials and 180º on others before searching the back quadrants, the position of the target-rich region relative to the participant after reorienting will not be consistent across trials, even when its position in the room remains constant. Because LPL may depend on a viewer-centered representation of space (Jiang and Swallow 2013; Jiang et al. 2014; Smith et al. 2010), the lack of consistent spatial coding introduced by spatial reorienting could impair LPL for target-rich regions behind the participant.

Previous studies have yet to explore the role of direction on LPL in 3D, large-scale search environments. In Smith et al. (2010), participants began search from one end of the room, meaning that the items never appeared behind them at the start of the trial. Participants in Jiang et al. (2014) began search in the middle of the outdoor space, yet that study did not report the effect of direction on LPL. Other studies have tested the role of direction on contextual cueing, a form of learning in which the distractor layout is predictive of the target’s location (Marek and Pollmann 2020; Shioiri et al. 2018). However, contextual cueing is distinct from LPL (Sisk et al. 2019), so the findings are not directly applicable to understanding the question at hand.

Thus, the present study investigated the acquisition of LPL in a virtual reality 3D space, where targets can appear frequently in front of or behind participants, in the absence of rich visual environmental cues. Participants searched for a letter T among letter Ls on the floor of a large nondescript virtual room with sparse visual cues. Participants began each trial at the center of the square room, facing the same direction in the virtual space. They pressed a key on a handheld controller to begin each search trial. After the start key was pressed, distractor Ls and the target letter T appeared on the floor of the search space. Participants searched until they found the letter T, pressing another button on the controller when they identified the target. After identifying the target, they indicated whether the T was light gray or dark gray using their controller. During training, the target appeared in one quadrant of the floor on half of the trials, with that target-rich quadrant being counterbalanced across participants. This allowed us to assess whether participants could acquire LPL in a visually sparse 3D space, and whether learning differed depending on whether the target-rich regions appeared in front of or behind the participants.

## Method

### Participants

College students between the ages of 18 and 25 years participated in this study in exchange for extra course credit. All participants had normal or corrected-to-normal visual acuity and were naive to the purpose of the study. The protocol was approved by the University of Minnesota Institutional Review Board.

Sample size was guided by previous studies on LPL, with typical sample sizes ranging from 8 to 24 participants (Jiang et al. 2013, 2014). Based on the effect size of *η*_*p*_^2^ = 0.63 (Jiang et al. 2014), G-power analysis suggests that a minimum of 4 participants were needed to reach a power of 0.95 with an alpha level of 0.05 (two-tailed). We therefore tested 16 participants.

Participants included 11 women and five men with a mean age of 19.7 years.

### Equipment

Stimuli were presented in an HTC Vive. The Vive weighs 470 g and is equipped with two OLED displays, each presenting a 1080 × 1200 pixel image to each eye, with partial stereo overlap. The combined image content is presented over a field of view that is approximately 112° horizontally and 116° vertically (Murphy et al. 2018). The Vive refreshes images about every 11 ms (90 Hz). The position and orientation of the Vive HMD is tracked at a rate of 120 Hz (Kreylos 2016) with < 22 ms latency (Niehorster et al. 2017) using a hybrid inertial and optical tracking system.

The virtual environment and experimental procedure were created using Unreal Engine version 4.22.

### Materials

A virtual environment designed to simulate a 30ft by 30ft square room was presented in the Vive headset. Each of the four walls were gray, and the floor was a concrete texture in a gray color. The texture of the floor created visual noise to simulate naturally occurring noise in real-world search environments. The floor texture was even in luminance across space, and shadows were not visible on the floor, in order to prevent uncontrolled modulations of search difficulty throughout the search space. The luminance of the four walls was based on an illumination model with a fixed angled light source from above, so the four walls were distinguishable, though there were no salient visual cues to associate with any of the four walls.

**Fig. 1:**
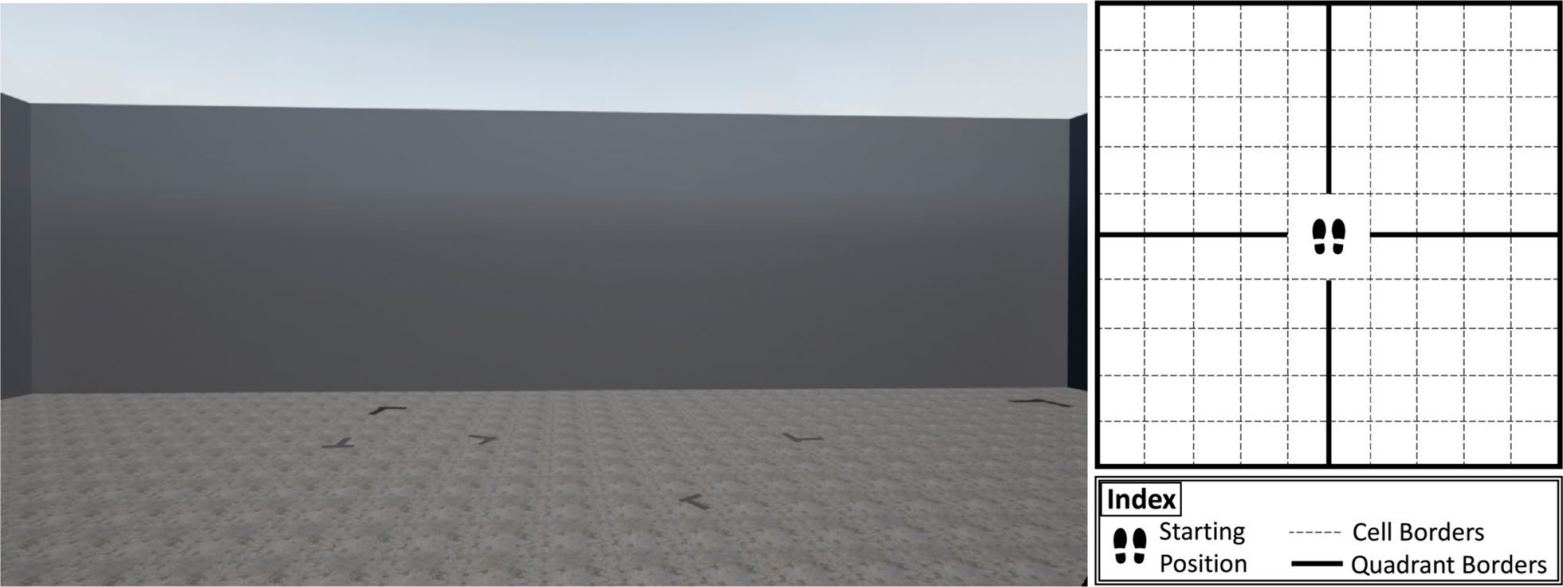
Left) an image of an example search trial with the target T in the upper left quadrant in a light gray color and distractor Ls in the upper right and upper left quadrants, some appearing in light gray and some in dark gray. Right) a schematic of the search space, with 96 possible target locations, excluding a 2 × 2 cell space immediately surrounding the participant’s starting spot. The same number of search stimuli appeared in each of the four quadrants outlined here (lines were not visible to participants)

The search stimuli consisted of a target letter T and distractor letter Ls. Half of the displays contained 11 Ls, half of them contained 15 Ls. In both cases the number of items appearing in each color in each quadrant was controlled, such that the same number of items appeared in each color, and no more than 2 items of the same color appeared in the same quadrant. The search space was divided into an invisible 10 × 10 grid, yielding 96 possible locations, excluding a 2 × 2 cell space immediately surrounding the participant’s starting spot (Fig. 1). The target and each distractor appeared in the center of one of these cells on each trial. The cell in which each stimulus appeared was random with the constraints that none of the stimuli overlapped, an equal number of stimuli appeared in each quadrant, and no stimuli appeared within a 2 × 2 space in the center of the search space. The final constraint prevented search stimuli from appearing directly beneath the participant’s starting position. The stimuli could appear in any orientation. The size of the stimuli were scaled with distance from the participant’s starting position—the farther a stimulus was from the center of the room, the larger it appeared. Specifically, the horizontal distance between the center of the room and each stimulus was calculated then multiplied by 3.5 before rendering. The stimuli could appear either in a light gray color or a dark gray color. The target’s color was randomized with the constraint that it appeared in each color on half of all trials. Each distractor’s color was randomized with the constraint that each quadrant had an equal number of search stimuli in each color.

### Design and procedure

Each trial began with the participant standing in the center of the search space, facing the same direction, both in the virtual world and in the real world. The researcher monitored each participant’s starting orientation on each trial and reminded participants to begin facing the correct direction if they were not consistently turning to face the starting wall before beginning search. Participants’ starting head direction was also measured, allowing for exclusion of trials without the correct starting orientation in head turn analyses. The researcher also monitored each participant’s starting position on the floor space. A large fixation cross on the floor of the virtual room enabled participants to determine the appropriate starting place on each trial if they walked around during search (though few participants strayed from their starting position during the experiment).

Once the participant was facing the starting direction—indicated by a temporary colored highlighting of the wall participants were to face—the participant began the search trial by pressing a button on their controller. Upon that button press, the highlighting of the starting wall disappeared and the search stimuli appeared on the floor of the search space. A ding also provided audio feedback indicating the beginning of the search trial. Participants were told to find the letter T as quickly as possible and to press a button on their controller as soon as they recognized the target. This stopped the timer. After that button press, the search stimuli disappeared. Participants then were instructed to indicate the color that the letter T had been when they found it: If it was light gray, they pressed their left controller key, and if it was dark gray, they pressed their right controller key. Participants received feedback on each trial in the form of a buzzer sound on incorrect trials.

Before beginning the experiment, participants read instructions appearing in their virtual environment, practiced using each of the controller buttons that was used in the experiment, and completed eight practice trials. Participants were welcome to complete more practice trials if they requested additional practice. Following practice, participants completed ten 24-trial blocks. Participants received enforced breaks after blocks 4 and 7, where they were required to remove the virtual reality headset. Participants were also allowed to take breaks as needed between trials or between other blocks. This minimized the effects of the fatigue that extended exposure to virtual reality can induce. Participants were free to move throughout the search space while searching, though most participants chose to simply turn in place while searching for the target. The experimenter monitored participants throughout the task to ensure they did not trip over the cord connecting the headset to the computer, they did not run into any obstacles in the real world, and they continued to follow instructions and turn to face the starting wall before starting a new search trial. Because participants rarely walked around during the experiment, they almost never had to be instructed to avoid real-world obstacles. However, as participants turned, the cord connecting the headset to the computer frequently wrapped around their legs. Most participants voluntarily unwound themselves between trials or stepped over the cord, though some required frequent reminders between trials from the experimenter. Participants had 15 s to complete each trial. After 15 s, the target increased in size, so participants could easily identify it and complete the trial. This time limit was chosen to minimize the fatigue experienced by participants during excessively long search trials.

Participants first completed two blocks of exposure, where they participated in the task, searching for the letter T and reporting its color, and the target was equally likely to appear in each of the four quadrants in the search space. Next, participants completed six training blocks, where on each trial, the target had a 50% chance of appearing in one high-probability, or target-rich quadrant. The target had about a 16.7% chance of appearing in each of the three target-sparse, low-probability quadrants. After the six training blocks, participants completed two testing blocks, where the target was equally likely to appear in each of the four quadrants. Participants were not informed about the target’s spatial probability distribution. The location of the target-rich quadrant (in front and to the left, in front and to the right, behind and to the left, or behind and to the right) was counterbalanced across participants.

Once all experimental trials were complete, participants answered a series of questions to probe awareness. Participants were asked whether they thought the target appeared in one area more often than in other areas. Then, regardless of their answer to the first question, laser pointer beams appeared, stemming from the participants’ controllers. They were asked to point the end of the laser beam at the location on the floor where they thought the target most often appeared. Finally, participants verbally rated on a scale of 0–4 their confidence in their choice of frequent target location.

## Results

### Accuracy

Mean accuracy was 90.8% (91.8% in the rich quadrant, 90.2% in the sparse quadrants, the difference was not significant, *t*(15) = 1.27, *p* = 0.22, Cohen’s *d* = 0.32). Errors included both search errors—where participants incorrectly identified a distractor as the target—and color discrimination errors—where participants found and identified the target but selected the incorrect color as the target color due to perceptual error. The two error types cannot be differentiated in the data because the color response also served as the target identification response. Since only one response was made, we cannot determine the precise rates of each error type. Participants with lower accuracy scores reported more difficulty in discriminating between light and dark gray than participants with higher accuracy scores. Inaccurate trials were excluded from further analysis.

### Reaction time (RT)

Trials lasting longer than 15 s were excluded from analyses (3% of all trials) because the target’s size was enlarged after 15 s of search time. Due to a coding error, the target was enlarged prematurely on a small number of trials, so those trials were also excluded (1% of all trials). Across all accurate trials under the 15-s cutoff, average RT was 4953 ms (*SE* = 200 ms). When the target appeared in one of the two quadrants in front of a participant, they were more likely to quickly identify the target because little or no head or body movement was required to bring the target into the field of view. For these trials with the target in front, average RT was 3967 ms (*SE* = 161 ms). When the target appeared behind participants average RT was 6009 ms (*SE* = 195 ms). RT for targets appearing in front was significantly faster than RT for targets appearing in back, *t*(15) = 15.89, *p* < 0.001, Cohen’s *d* = 3.97.

For all RT analyses, trial blocks of 24-trials each were pooled into two-block, 48-trial epochs. Epoch 1 includes the first two blocks in which probability distribution was even, meaning the target was equally likely to appear in each of the four quadrants. Epochs 2–4 include the six training blocks in which the target had a 50% chance of appearing in the high-probability quadrant on each trial and a 16.7% chance of appearing in each of the other three quadrants. Epoch 5 includes the final two testing blocks, in which the target was equally likely to appear in each of the four quadrants.

Because reaction time is much faster when the target appears in front of participants than when it appears behind them, we compared RT on trials where the target appeared in the target-rich quadrant only with RT on trials where the target appeared in the adjacent target-sparse quadrant that matched the rich quadrant in direction. In other words, if the rich quadrant was in front of a participant (e.g., in front and to the left), the sparse quadrant used for RT comparison would also be in front of the participant (e.g., in front and to the right). If the rich quadrant was behind a participant, the comparison would be made using trials where the target appeared in the one sparse quadrant that was also behind the participant.

**Fig. 2:**
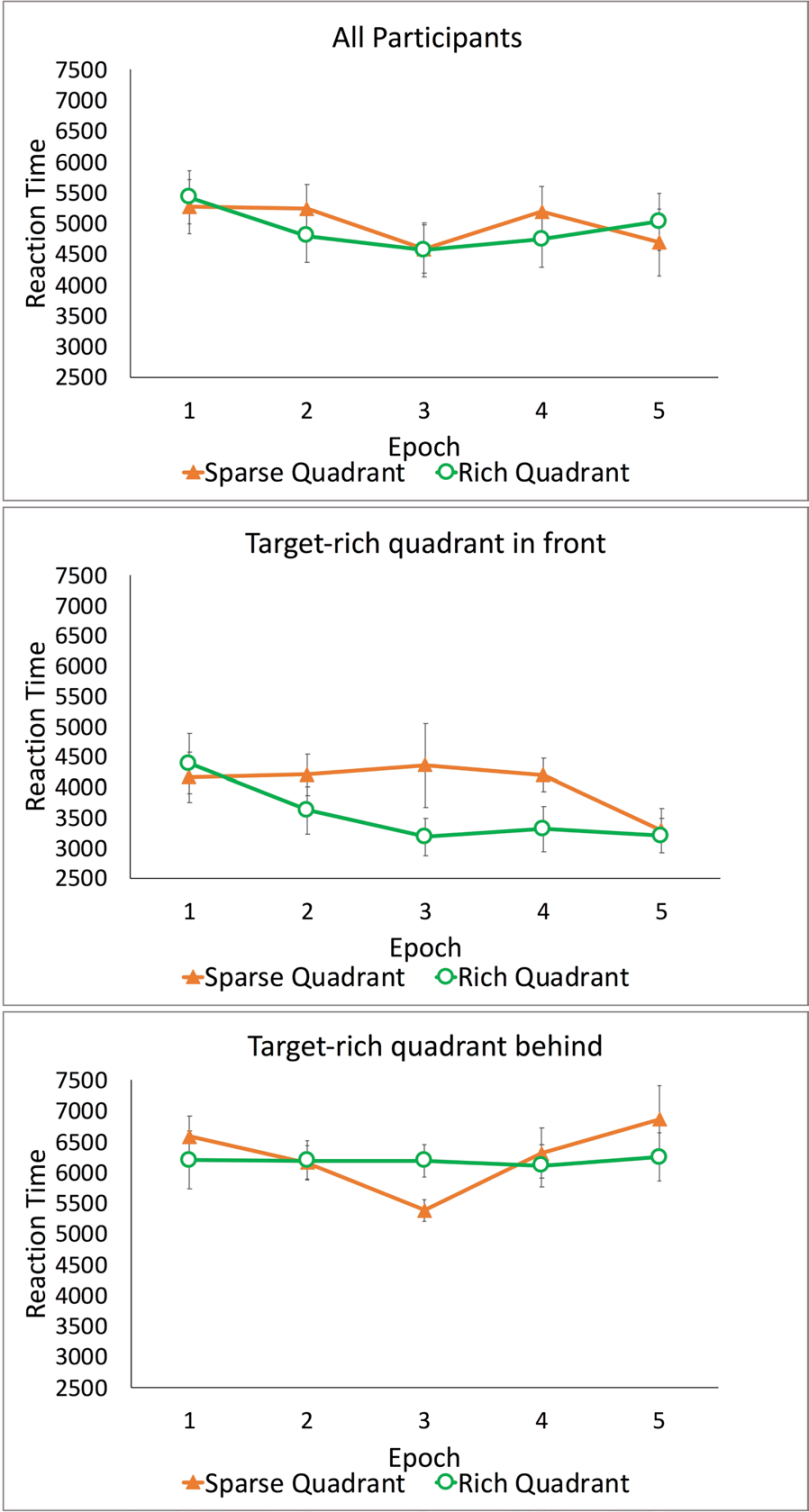
Average RT across five epochs, presented separately for trials in which the target appeared in the target-rich quadrant and trials in which the target appeared in the direction-matched target-sparse quadrant. Data is presented for all participants (top), then presented separately for participants whose target-rich quadrant was in front of them (middle) and for those whose target-rich quadrant was behind them (bottom). The rich quadrant is classified as the quadrant that the target was most likely to appear in during the training epochs 2–4. For epoch 1, the RT for the quadrant that would later become the rich quadrant is plotted as “rich,” and for epoch 5, the RT for the previously rich quadrant is plotted as “rich.” This allows for tracking of patterns of learning across changes in the probability distribution. Error bars represent ± 1 standard error of the mean

Data from all participants are shown in Fig. 2 (top). RT was numerically faster in the rich quadrant than in the direction-matched sparse quadrant. However, the pattern of results depended on whether the rich quadrant was in front of or behind the participant. For the participants with a rich quadrant in front of them, there is clear evidence of learning—faster RT in the rich quadrant (Fig. 2, middle). For the other participants with a rich quadrant behind them, there is no evidence of learning (Fig. 2, bottom). Marginal means for the data can be found in Table 1.

**Table 1:** Mean RT in milliseconds by condition

	Rich in front			Rich behind		
	Exposure	Training	Testing	Exposure	Training	Testing
Rich	4383	3370	3201	6202	6164	6250
Sparse	4164	4255	3285	6584	5952	6860

#### Exposure phase (Epoch 1)

The first two blocks (Epoch 1) did not include a target probability manipulation. Instead, they served to acclimate participants to the task before probability training began. As expected, RT did not differ between trials in which the target appeared in what would later become the target-rich quadrant and trials in which the target appeared in the adjacent quadrant that would later be defined as target-sparse in the training phase, *t* < 1.

#### Training phase (Epochs 2–4)

An ANOVA using quadrant (sparse or rich), training epoch (2–4), and direction of rich quadrant (front or back) as factors showed a main effect of direction, with faster RT for participants with a rich quadrant in front of them than those with a rich quadrant behind them, *F*(1, 14) = 52.00, *p* < 0.001, *η*_*p*_^2^ = 0.79. The main effect of quadrant was significant, *F*(1, 14) = 4.57, *p* = 0.05, *η*_*p*_^2^ = 0.25. The main effect of epoch was not significant, *F* < 1. Epoch did not interact with the direction of the rich quadrant, *F* < 1, or with quadrant, *F* < 1. However, the quadrant effect interacted with direction, *F*(1, 14) = 12.13, *p* = 0.004, *η*_*p*_^2^ = 0.46. The three-way interaction was not significant, *F*(2, 28) = 1.70, *p* = 0.20, *η*_*p*_^2^ = 0.11.

To understand the quadrant by direction interaction effect, we analyzed data separately for participants with a rich quadrant in front of or behind them. For participants with a rich quadrant in front of them, an ANOVA on target quadrant (rich or sparse) and training epoch (2–4) produced a significant main effect of quadrant, *F*(1, 7) = 15.48, *p* = 0.006, *η*_*p*_^2^ = 0.69, no main effect of epoch, *F* < 1, and no interaction, *F* < 1. Thus, there is clear evidence of an RT benefit in the rich quadrant, relative to the direction-matched sparse quadrant for participants with a rich quadrant in front of them.

For participants with a rich quadrant behind them, an ANOVA on target quadrant and training epoch showed no main effect of target quadrant, *F* < 1, no main effect of epoch, *F*(2, 14) = 1.51, *p* = 0.25, *η*_*p*_^2^ = 0.18, and no interaction between quadrant and epoch, *F*(2, 14) = 2.12, *p* = 0.16, *η*_*p*_^2^ = 0.23. Thus, there was no evidence of LPL in RT for participants with a rich quadrant behind them.

#### Testing phase (Epoch 5)

There was no evidence that LPL persisted in epoch 5 when the target was equally likely to appear in each quadrant. An ANOVA on RT in epoch 5 using target quadrant (previously rich vs. previously sparse) and direction (rich quadrant in front vs. rich quadrant in back) as factors found only a main effect of direction, *F*(1, 14) = 51.13, *p* = 0.001, *η*_*p*_^2^ = 0.79. There was no effect of target quadrant, *F*(1, 14) = 1.04, *p* = 0.33, *η*_*p*_^2^ = 0.07, and there was no interaction between quadrant and direction, *F* < 1. Follow-up tests showed no significant difference in RT between the previously rich and previously sparse quadrants for either participants with a rich quadrant in front, *t*(7) = 0.22, *p* = 0.83, or those with a rich quadrant behind them, *t*(7) = 1.08, *p* = 0.32.

The lack of persistence of LPL in the testing phase may suggest that the RT advantage observed during the training phase in participants with a rich quadrant in front was the result of repetition priming, rather than LPL. If this were the case, we would expect RT to be faster on trial N when the target appeared in the same quadrant on trial N as on trial N-1, regardless of whether the target appears in the rich quadrant. However, quadrant repeat trials (mean RT = 3534 ms, *SE* = 359) were not significantly faster than quadrant nonrepeat trials (mean RT = 3594, *SE* = 223). To confirm this, we analyzed data from the participants with a rich quadrant in front of them. An ANOVA on RT in the training phase, where we did see an LPL effect for these participants, with quadrant repetition and quadrant condition (rich or sparse) as factors showed no main effect of quadrant repetition, *F* < 1. Furthermore, when the analysis was restricted to trials in which the target’s quadrant differed from the preceding trial’s target quadrant, we continued to observe an LPL in participants whose rich quadrant was in front of them, *t*(7) = 6.27, *p* < 0.001, Cohen’s *d* = 2.22, with faster RT when the target appeared in the rich quadrant (*M* = 3191 ms, *SE* = 249) than when it appeared in the sparse quadrant (*M* = 4300 ms, *SE* = 216).

##### First head turn

**Fig. 3:**
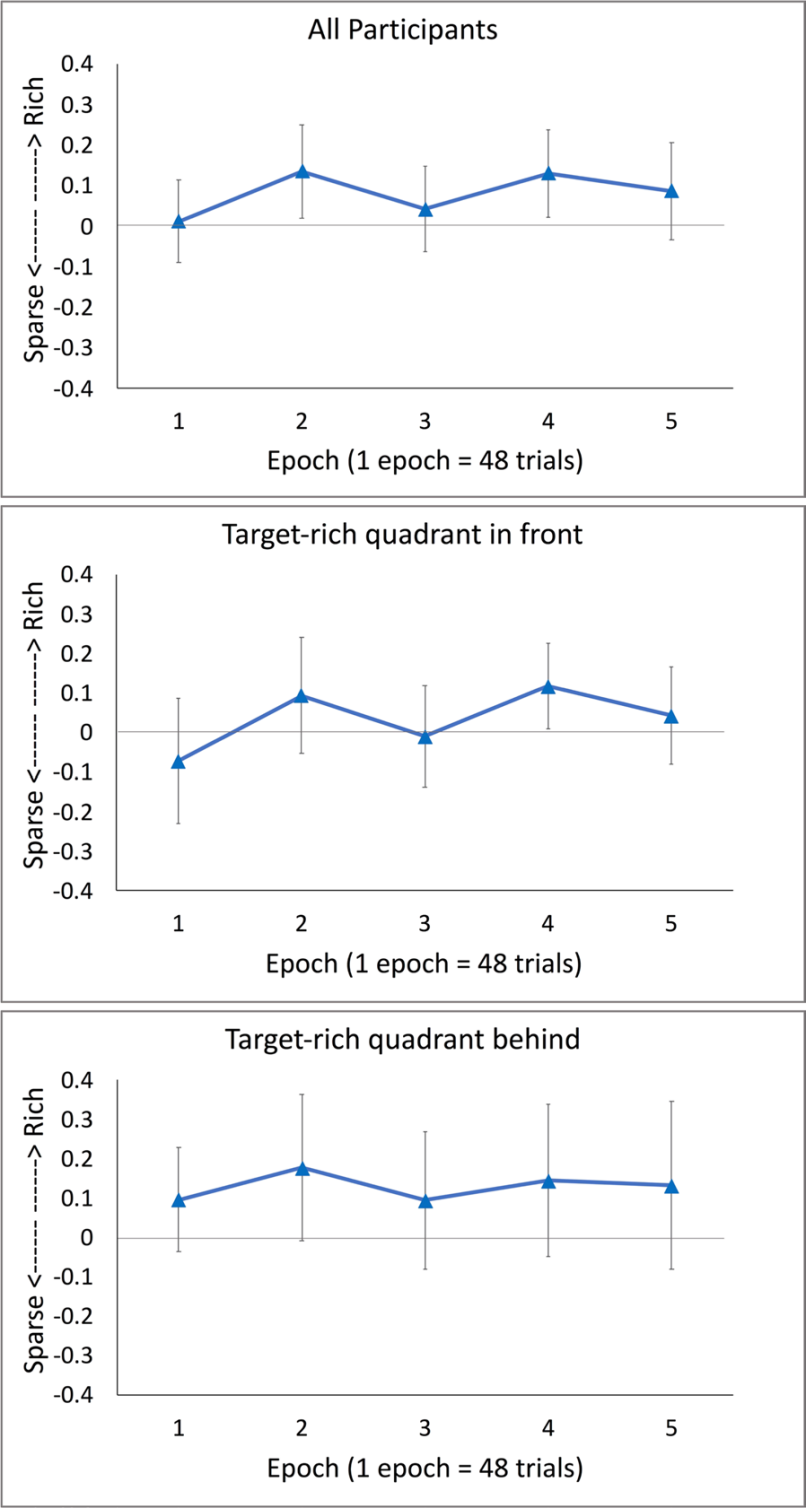
Average direction of first head turn across trials within an epoch for all participants (top), for participants with a target-rich quadrant in front of them (middle), and for participants with a target-rich quadrant behind them (bottom). Positive values indicate a tendency to turn toward the target-rich quadrant, negative values indicate a tendency to turn away from the target-rich quadrant, and values near 0 indicate the lack of a consistent tendency to turn clockwise or counterclockwise at the start of a search trial. Bars represent standard error of the mean

The position of participants’ headsets in degrees along the *x*-, *y*-, and *z*-axes were output every 30 ms during the first two seconds of search. Participants were not given instructions regarding which direction to turn first during search, so on any given trial, they could turn their head to the left or to the right. We measured the direction of the first head turn by first identifying a time point at which the velocity of the head turn along the *z-*axis, or the azimuth, exceeded 20-deg/sec. Mean latency of the first head turn was 430 ms (*SE* = 31) overall. Mean latency on trials in which the target appeared in the rich quadrant was 425 ms (*SE* = 31), and mean latency on trials in which the target appeared in the sparse quadrant was 435 ms (*SE* = 31). There was not a significant difference in latency of the first head turn between rich and sparse trials, *t*(15) = 1.16, *p* = 0.26, Cohen’s *d* = 0.29. We then compared the degrees on that plane at that time point to the starting value to calculate the direction—left or right—of the participant’s first head turn. Then, we determined whether the target-rich quadrant was to the left or the right of each participant and coded each head turn as either a turn toward the rich quadrant (+ 1) or a turn away from the rich quadrant (−1). If there is no bias in the direction of head turns, the mean should be 0. A tendency to turn toward the rich quadrant should result in positive values (mean greater than 0). This analysis includes all trials, not just correct trials under 15 s. All trials were included because this analysis only considers the first 2000 ms of each trial in which the initial head turn is likely to occur. Trials in which participants take longer than 15 s to find the target or trials in which participants eventually make an incorrect response are unlikely to systematically differ from correct trials within the first 2000 ms of search. The same analysis excluding incorrect trials and trials over 15 s yielded the same pattern of results. Data are visible in Fig. 3.

First, it is worth noting that at the start of the experiment, participants did not demonstrate a preference toward turning in a particular direction. The average direction of the first head turn across all of the trials in Epoch 1 (*M* = 0.01, *SE* = 0.10) was not significantly different from 0, *t* < 1, meaning that participants turned both toward and away from the target-rich quadrant with equal frequency at the start of the experiment. Second, participants also did not develop a preference of turning toward the rich quadrant over time. The mean across all epochs (*M* = 0.08, *SE* = 0.10) was not significantly greater than 0, *t* < 1, and neither the mean of any individual epoch nor the mean of the training epochs differed significantly from 0 or from the mean of epoch 1, lowest *p* = 0.08. The same was true when analysis was limited to participants with the rich quadrant in front of them. An ANOVA on head turn data in the training phase with epoch as a within-subjects factor and direction of the rich quadrant (front or back) did not yield a significant effect of epoch, *F*(2, 28) = 1.53, *p* = 0.24, *η*_*p*_^2^ = 0.10, nor an interaction between epoch and direction of the rich quadrant, *F* < 1.

##### General direction learning

While only participants with a target-rich quadrant in front of them displayed evidence of location probability learning in search RT, it is possible that participants with a target-rich quadrant behind them developed a more general bias. Specifically, they may have learned that the target appeared more often behind them, even if they could not learn that the target appeared to their back-left or back-right specifically. An ANOVA on RT with epoch (1–5) and the target’s general location (front or back) as within-subject factors and location of the rich quadrant (front or back) as a between-subjects factor found only a significant main effect of target location (slower response to targets appearing behind a participant), *F*(1, 14) = 269.72, *p* < 0.001, *η*_*p*_^2^ = 0.95, with no interaction between target location and location of the rich quadrant, *F*(1, 14) = 1.14, *p* = 0.30, *η*_*p*_^2^ = 0.08. This suggests that participants with a rich quadrant behind them did not show a significantly smaller RT cost when targets appeared behind them than participants with a rich quadrant in front of them. The cost, or the difference in RT between targets appearing behind and targets appearing in front of participants, was numerically smaller for those with a rich quadrant behind them (1906 ms) than for those with a rich quadrant in front of them (2178 ms), but this effect was not significant, *t*(14) = 1.06, *p* = 0.31, Cohen’s *d* = 0.53.

When considering only the biased training phase (epochs 2–4), the interaction between the target’s general location and the direction of the rich quadrant reached marginal significance, *F*(1, 14) = 3.80, *p* = 0.07, *η*_*p*_^2^ = 0.21. However, this effect disappeared when only considering trials in which the target appeared in a different quadrant on trial *N* than it had on trial *N* − 1, *F* < 1. This suggests that any advantage for those with a rich quadrant behind them for targets behind them was likely due to repetition priming effects.

When looking at general leftward or rightward biases that disregard front-back differences, these same analyses yielded no significant effects, suggesting that no general directional biases were learned for those with the target-rich quadrant behind them.

##### Set size

Previous studies on LPL in 2D search have shown that probability learning not only decreases RT for trials where the target appears in the target-rich quadrant, but it also reduces the search slope for those target-rich trials. Because our study included set sizes 12 and 16, we examined whether search slope was shallower in the rich quadrant (see Table 2). This analysis was restricted to the group that showed LPL in RT, using data from the three training epochs where the effect was observed. An ANOVA on RT with set size and target quadrant as factors showed a significant main effect of quadrant, *F*(1,7) = 18.18, *p* = 0.004, *η*_*p*_^2^ = 0.72, but no significant effect of set size, *F* < 1, and no interaction between set size and quadrant, *F* < 1. Thus, we did not find evidence that LPL reduced search slope. The lack of a search slope effect might mean that LPL in 3D search environments did not enhance search efficiency. However, it is possible that we have missed a slope effect given the small number of observations in the target-sparse condition (averaging 11 trials per participant in each condition). Future studies are needed to determine whether the mechanism of LPL in 3D search environments involves increased search efficiency.

**Table 2:** Mean RT in milliseconds by condition

	Rich in front		Rich behind	
	Set size 12	Set size 16	Set size 12	Set size 16
Rich	3224 (*n* = *35*)	3587 (*n* = 31)	5697 (*n* = 29)	6439 (*n* = 33)
Sparse	4087 (*n* = 11)	4132 (*n* = 11)	6129 (*n* = 11)	5921 (*n* = 9)

This table includes data from the training phase of the experiment. The number of observations differs across conditions. The average number of observations under each condition is indicated parenthetically and labeled as *n*

##### Awareness

When asked whether the target appeared in some places more often than others, nine out of the 16 participants answered yes. Of those nine, five correctly identified the target-rich quadrant. Two others who initially said the target was evenly distributed throughout the search space also correctly guessed the target-rich quadrant. Of the seven who correctly guessed the rich quadrant, three had had a rich quadrant in front of them, and four had had a rich quadrant behind them.

Confidence ratings were low for all participants, regardless of whether or not they correctly identified the target quadrant. On a scale of 1–4 with 1 being “completely guessing” and 4 being “absolutely certain,” the overall group average rating was 2.0, and those who guessed correctly were slightly less confident in their choice of target quadrant (average confidence of 1.86) than those who guessed incorrectly (average confidence of 2.11). There was no significant difference in the magnitude of LPL (the RT difference between the sparse and the rich trials) between those who correctly guessed the rich quadrant and those who did not*, t*(14) = 1.82*, p* = 0.09, Cohen’s *d* = 0.91. The pattern of visual search results reported above remain unchanged when these seven participants’ data were excluded. Owing to the small sample size and the single-item nature of the recognition test, caution should be exerted in interpreting the explicit recognition data (Vadillo et al. 2020). Compared with previous LPL studies, the proportion of participants correctly identifying the high-probability quadrant in the current study—44%—was comparable to that observed in small-scale search studies in 2D environments (Jiang et al. 2018), and much lower than the proportion observed in previous large-scale search studies in 3D environments (~ 70% in Jiang et al. 2014 and nearly 90% in Smith et al. 2010). Additional testing is needed to further characterize the explicit versus implicit nature of learning in 3D environments with sparse visual cues.

##### Return to Jiang et al. (2014)

Previous studies on LPL in 3D environments did not examine the effect of direction. To determine whether the current finding was unique or whether direction also mattered in previous studies, we re-analyzed the data from the outdoor search task of Jiang et al. (2014). Specifically, we tested whether or not participants with a target-rich quadrant behind them showed learning in an outdoor environment with many complex visual cues and landmarks. As in the present study, half of the 16 participants in Experiment 1 of Jiang et al. (2014) had the target-rich quadrant in front of them at the start of a trial. During the training blocks, these eight participants exhibited a significant LPL effect, *F*(1, 7) = 9.06, *p* = 0.02, *η*_*p*_^2^ = 0.56. In contrast, the LPL effect failed to reach statistical significance in the training blocks for the eight participants whose target-rich quadrant appeared behind them, *F*(1, 7) = 3.02, *p* = 0.13, *η*_*p*_^2^ = 0.30. Numerically, LPL was about twice as large (~ 2000 ms) for participants with a target-rich quadrant in front of them than for participants with a target-rich quadrant behind them (~ 900 ms). This exploratory analysis hints at the possibility that even with rich visual cues, LPL in 3D search spaces may be asymmetrical, favoring space within a participant’s field of view at the start of search.

Although Jiang et al. (2014) and the present study are methodologically distinct, there are enough similarities in the task parameters that it may be informative to strengthen the power of the analyses by combining the two datasets. The number of blocks and epochs was not the same, so we averaged RT across all training blocks in each experiment. An ANOVA on RT with quadrant (rich or sparse) as a within-subjects factor and direction of the rich quadrant (front or back) as a between-subjects factor found both a significant effect of quadrant, *F*(1, 30) = 14.73, *p* = 0.001, *η*_*p*_^2^ = 0.33, and a significant interaction between quadrant and direction of the rich quadrant, *F* (1, 30) = 5.74, *p* = 0.02, *η*_*p*_^2^ = 0.16. Considering only participants with a rich quadrant behind them, there was no significant effect of quadrant, *t*(15) = 1.62, *p* = 0.13, Cohen’s *d* = 0.40. Thus, doubling the sample size by combining these two studies still did not yield significant location probability learning in participants with a rich quadrant behind them.

## Discussion

In this large-scale 3D search environment devoid of complex visual cues, participants did show evidence of LPL, but only when the target-rich quadrant was in front of them, relative to their starting position. These findings demonstrate, on the one hand, the robust nature of LPL in large-scale visual search environments. On the other hand, they highlight a theoretically relevant limitation of LPL: Learning may be disrupted for information appearing outside of the initial field of view. Because participants whose rich quadrant was behind them had to frequently turn around to find the target, and because the direction and degrees of that turn were not entirely consistent from trial-to-trial, they were unable to accurately update and maintain statistical information about the target’s location across many trials. This suggests that consistent search patterns are necessary for LPL to occur in large-scale 3D environments in the absence of rich environmental visual cues.

Previous studies involving large-scale search in 3D outdoor environments did observe faster reaction time on trials where the target appeared in the target-rich quadrant than on trials where it appeared in a target-sparse quadrant across the entire pool of participants (Jiang et al. 2014). Further scrutiny of the data from Jiang et al. (2014) following the findings presented here revealed a previously unnoticed distinction between participants with a target-rich quadrant in front of them and participants with a target-rich quadrant behind them. Similar to the present findings, the participants in Jiang et al. (2014) showed a significant LPL effect during training only when the rich quadrant appeared in front of them.

The pattern of RT results is analogous between Jiang et al. (2014) and the present study, but there are some interesting differences in the pattern of the head turn data. In Jiang et al. (2014), participants developed a habit of turning their head in the direction of the target-rich quadrant over the course of the experiment. Although we observed LPL in RT in participants with a rich quadrant in front of them, those participants did not tend to turn their head in the direction of the rich quadrant. The exact reasons for the difference in head turn results are unclear, but several factors may have contributed to the findings. First, the search stimuli were different between the two studies. Jiang et al. (2014) asked participants to search for a single coin on the ground. Although the coin was small and difficult to find within the visual noise of the concrete ground, it did not involve search for a target among discrete distractors as in the present study. This may have changed search behavior. In fact, the latency for the first head movement was considerably longer in the outdoor task (Won et al. 2016; mean 561 ms) than in the current study (~ 430 ms). It is possible that participants in the outdoor task turned their heads after they had spotted the target on some trials.

Another notable distinction between participants’ behavior in Jiang et al. (2014) and the present study that could have influenced head turns is that participants actively walked around the search space in Jiang et al. (2014), while they remained mostly stationary in the present study. In both cases, participants were allowed to wander throughout the search space, but participants rarely did so in VR—they tended to rotate, rather than walk around during search, as is frequently observed in VR contexts (Pausch et al. 1996). The tendency to remain stationary, and the evident lack of navigational planning, may have precluded the formation of certain motor-related habits. Experimentally induced movement, even in terms of required rotation or navigation, may increase the likelihood that statistical learning effects would be expressed in the pattern of head turns at the start of a search trial. Future research should be done to identify the factors that lead to expression of LPL in head-turn tendencies, as opposed to or in addition to RT effects.

Another distinction between the present study and prior studies on LPL is the lack of an RT bias for the high-probability quadrant in the testing phase. Might the lack of persistence indicate a lack of probability learning, with the bias observed in the training phase reflecting repetition priming effects? Further analysis suggests that repetition priming cannot explain the quadrant effects. In our study, the bias in the training phase existed independently of repetition priming effects, with the effect persisting when quadrant-repeat trials were removed. The lack of the effect in the testing phase then is likely attributable to one of two other factors. First, the relatively small number of training trials may have reduced the persistence of LPL. Typically, LPL studies include hundreds of training trials. Due to fatigue effects caused by virtual reality, the present study was limited to 144 training trials. This may have made the bias more susceptible to interference from the new, even statistical distribution in the testing phase.

Second, in typical computerized LPL studies, search can be accomplished using only oculomotor movements. In the present study, search required both oculomotor movements and head movements, with the possibility of body movements as well. This may increase the difficulty of maintaining search biases in the absence of reinforcement, as procedural habits across many different systems must be maintained for an RT bias to persist. This may also have made it more difficult to detect search biases in RT alone. Considering these potential influences alongside the lack of dependence on repetition priming, our findings in the training phase likely do represent statistical learning, with the effect simply being more fragile and susceptible to interference in the testing phase than is typically observed in other LPL studies with longer training and simpler computerized search.

Some previous studies have observed spatial statistical learning in 3D environments, but these studies examined contextual cueing, rather than LPL (Marek and Pollmann 2020; Shioiri et al. 2018; Zang et al. 2017). Shioiri et al. (2018) tested contextual cueing in 3D by asking participants to sit in the center of a circle of six monitors, each of which contained search stimuli. Participants were asked to find the letter T among letter Ls, and the search arrays repeated on some trials. On those repeated arrays, the positions of all of the distractors on all of the monitors and the target position repeated. As in previous research on contextual cueing in 2D, participants were faster at finding the target on repeated displays than on novel displays, showing that attention was guided by the learned association between the distractor array and the target location. Crucially, this RT benefit of display repetition was observed even for displays where the target appeared behind the participant, outside of their initial field of view.

Marek and Pollmann (2020) took this line of experimentation further and tested contextual cueing in 3D virtual environments, much like we have done in the present study on LPL. Like Shioiri et al. (2018), Marek and Pollmann observed a contextual cueing effect that was present even when the target was outside of the initial field of view. Although the studies that observed intact contextual cueing in 3D search spaces did use simple stimuli that controlled for the influence of visual complexity, contextual cueing represents a form of visual statistical learning that may be mechanistically distinct from LPL (see Sisk et al. 2018, 2019). Contextual cueing involves learning of associations between the locations of distractors and the target’s location, whereas LPL involves repeated shifts of attention to one region of space across many search trials. The distinction between the two types of visual statistical learning is particularly relevant in the context of a transition to a 3D search space. Contextual cueing, which relies on memory for the relative positions of the distractors and target, may be particularly robust to changes in the local search space that occur as one turns and reorients during search. This is because regardless of one’s orientation, the association between the distractor array and the target location is maintained. LPL, on the other hand, requires a consistent search pattern that may be disrupted by the turning and reorienting that occurs during search in 3D environments.

The present findings support this possibility, demonstrating an absence of LPL in participants who frequently had to turn around and reorient in order to find the search target. It is worth noting that both participants with a target-rich quadrant in front and those with a target-rich quadrant in back had to turn around during search frequently, so reorientation during search does not inherently interfere with LPL. However, participants with a rich quadrant behind them had to reorient more frequently, and more crucially, they had to reorient before finding the target and coding its location. Participants with a rich quadrant in front of them often found and identified the target without having to turn and reorient, so its coding was consistent—if the target-rich quadrant was to their upper right when they began the trial, it was still to their upper right when they found the target. Participants with a rich quadrant in back, however, had to turn their bodies before finding the target in the rich quadrant, since the rich quadrant was not within their starting fields of view. If the target-rich quadrant were behind them and to the right, they may sometimes turn 90° to the right and find the target in the rich quadrant in a position that is in their upper right visual field. Other times, however, they may turn 180º to the left and find the target in the rich quadrant in a position that is in their upper left visual field. Thus, it is not the simple frequency with which one reorients across all search trials that disrupts LPL—it is primarily the inconsistency of the rich quadrant’s position relative to the participant when they find the target there. If participants were required to turn in the same direction on every search trial, we may expect those with rich quadrants behind them to show an emergence of LPL. However, the lack of LPL in the testing epoch when the probability distribution was even, even in participants with a target-rich quadrant in front of them, may suggest that the frequency of reorienting does interfere with expression of LPL after learning has occurred.

Alternatively, some evidence suggests that the lack of learning in those with a rich quadrant behind them is related to the tendency to rely on perception, rather than memory, in search tasks in which some items appear outside of the initial field of view. Oliva et al. (2004) found that in panoramic search, participants tended to search among visible objects first, rather than using memory for the location of objects in familiar environments to guide them directly to the search target that was out of the initial field of view. Other studies have also found that participants tend to minimize reliance on memory during search, in favor of perceptually guided search (Ballard et al. 1995; Droll and Hayhoe 2007; O’Regan 1992).

This tendency to rely on perception before resorting to memory may interfere with either the acquisition or expression of LPL in those with a rich quadrant behind them. Searching for the target when it frequently appears behind a participant may interfere with the coding of the location of the rich quadrant, making it more difficult to acquire LPL. It may also interfere with the expression of LPL, as participants may be slowed by their proclivity for searching for the target in front of them first and their over-reliance on potentially misleading visual cues, effectively masking the LPL effect. This may explain why those with a rich quadrant behind them were numerically slower at finding the target when it appeared behind them than those with a rich quadrant in front of them. It may also have contributed to the lack of an LPL effect in the testing phase, whereby even those who had been trained with a rich quadrant in front of them were slowed by their tendency to rely on visual information, rather than memory. However, those with a rich quadrant in front of them did appear to rely on memory during search, as demonstrated by the significant LPL in that group, so search was not entirely guided by perceptual cues, even in the group that frequently had access to a perceptual view of the target. Regardless, this tendency to rely on perception, rather than memory, represents an additional computational challenge to LPL in 3D search environments where targets can appear either in front of or behind one’s starting position.

Using a controlled, visually sparse 3D search space, the present study provides important insights into the mechanisms underlying LPL in real-world search environments by controlling for the influence of rich environmental cues. These findings show the importance of consistent egocentric coding and consistent search patterns in acquiring and expressing LPL. This suggests that attempts to harness the robust, powerful nature of spatial statistical learning in real-world search scenarios may be limited by the locations of frequent target locations, consistency of search direction, and informed, consistent use of complex environmental cues. Future work must be done to establish a set of necessary and sufficient conditions for LPL to occur in large-scale, 3D search environments and to determine the conditions under which training in one setting can transfer to another. The present study provides a baseline by which to compare the effects of added visual cues, forced consistent search patterns, and changes in the characteristics of the search space.

### Next steps

The empirical scope of this paper is limited by the necessity of in-person data collection for search in virtual reality and the impossibility of conducting in-person testing during the ongoing COVID-19 pandemic. However, we believe this paper provides an important baseline that will be useful to researchers exploring this new genre of visual search in large-scale 3D environments once in-person testing can resume.

First, future studies should explore the influence of varied starting positions and orientations on the effects observed here. Before in-person testing was halted, we collected preliminary data in two conditions: one in which the participant’s starting orientation changed randomly from trial to trial while the rich quadrant’s location in room coordinates remained constant (allocentric learning condition) and one in which the participant’s starting orientation changed while the rich quadrant’s location relative to the starting orientation on each trial remained constant (egocentric learning condition). While we have been unable to collect enough data to report in a published manuscript, our data thus far showed evidence of learning in the egocentric learning condition, but only for those with a target-rich quadrant in front of them. No evidence of learning was observed in the allocentric learning condition. This finding suggests that rich locations may be coded in an egocentric reference frame, though further data collection is needed to confirm this finding.

Second, future research should explore the influence of rich visual environmental cues on learning. One potential reason for why we did not observe learning for those with a rich quadrant behind them in the present study but Jiang et al. (2014) did find a larger (though still not significant) effect in that group could be the presence of rich visual environmental cues in the latter study. We created an experiment that is identical to the one presented here except for the addition of a visually rich environment surrounding the search space. The data from four participants resemble the findings observed here, with no evidence of learning in the group with the rich quadrant behind them. However, during testing it became clear that participants did not pay attention to the visual environment, as it simply surrounded the search space and did not have any bearing on search itself. In fact, some participants noted more than halfway through testing that they had just realized that there was a visual environment surrounding the floor of the search space. It will be important to create experimental settings in which the visual details surrounding the search space are integrated with the search space, and not automatically filtered out.

Finally, future research should further enhance the 3D nature of search. In the present experiment, all search items appeared on the floor. Thus, although the search environment was 3D, and search required participants to search behind them, the locations of search items only varied across two dimensions. It will be important to conduct an experiment where the locations of search items vary across all three dimensions, appearing at different heights as well as at different locations across the ground plane.

While inclusion of these planned experiments in the present report would have been ideal, we believe that this study is important and potentially influential in establishing a baseline on which these future experiments can build. Location probability learning is a powerful effect that is robust to aging, cognitive load, and serious neurological disorders. It therefore has the potential to greatly improve search efficiency in many real-world search tasks, if the proper training can be provided. In order to utilize location probability learning in real-world search, however, researchers must first understand i) the capacity of the effect in 3-dimensional search environments and ii) the factors that modulate this kind of visual statistical learning in 3-dimensional search environments. The present study provides a crucial baseline by exploring location probability learning in 3-dimensional search spaces while controlling for confounding factors like complex visual cues or landmarks. The observation that location probability learning is weaker when targets most often appear behind a person is critical in considerations of location probability learning in real-world contexts, as many real-world search contexts involve search of space outside of one’s initial field of view. Future research may focus on identifying the factors that allow participants to overcome this limitation of location probability learning while maintaining the implicit, habitual nature of location probability learning that likely underlies its resistance to aging, cognitive load, and neurological disorders.

## Data Availability

Data in aggregate form will be made available to other researchers upon request. Individual raw data cannot be made available or added to a repository due to restrictive language in the confidentiality section of the consent form.

## Footnotes

1 In virtual reality research, the term *field of view* is typically contrasted with the term *field of regard*, with the latter referring to the entire virtual space and the former referring to the area of that space that is within a participant’s view. Here, we use field of view to refer to the region of space in front of participants that is visible without the need for body movements.

## References

[CR1] Awh E, Belopolsky AV, Theeuwes J (2012). Top-down versus bottom-up attentional control: A failed theoretical dichotomy. Trends in Cognitive Sciences.

[CR2] Ballard DH, Hayhoe MM, Pelz JB (1995). Memory representations in natural tasks. Journal of Cognitive Neuroscience.

[CR3] Droll, J. A., & Hayhoe, M. M. (2007). Trade-offs between gaze and working memory use. *Journal of Experimental Psychology. Human Perception and Performance*, *33*(6), 1352–1365. https://doi.org/10.1037/0096-1523.33.6.1352 10.1037/0096-1523.33.6.1352 18085948

[CR4] Druker M, Anderson B (2010). Spatial probability aids visual stimulus discrimination. Frontiers in Human Neuroscience.

[CR5] Geng JJ, Behrmann M (2002). Probability cuing of target location facilitates visual search implicitly in normal participants and patients with hemispatial neglect. Psychological Science.

[CR6] Itti L, Koch C (2001). Computational modelling of visual attention. Nature Reviews. Neuroscience.

[CR7] Jiang YV (2018). Habitual versus goal-driven attention. Cortex.

[CR8] Jiang YV, Sha LZ, Sisk CA (2018). Experience-guided attention: Uniform and implicit. Attention, Perception, & Psychophysics.

[CR9] Jiang YV, Sisk CA (2018). Habit-like attention. Current Opinion in Psychology.

[CR10] Jiang YV, Swallow KM (2013). Spatial reference frame of incidentally learned attention. Cognition.

[CR11] Jiang, Y. V., Swallow, K. M., & Rosenbaum, G. M. (2013). Guidance of spatial attention by incidental learning and endogenous cuing. *Journal of Experimental Psychology. Human Perception and Performance*, *39*(1), 285–297. https://doi.org/10.1037/a0028022 10.1037/a0028022 PMC3431435 22506784

[CR12] Jiang YV, Won B-Y, Swallow KM, Mussack DM (2014). Spatial reference frame of attention in a large outdoor environment. Journal of Experimental Psychology: Human Perception and Performance.

[CR13] Marek, N., & Pollmann, S. (2020). Contextual-cueing beyond the initial field of view: A virtual reality experiment. *Brain Sciences*, *10*(7). https://doi.org/10.3390/brainsci10070446 10.3390/brainsci10070446 PMC7407752 32668806

[CR14] Miller J (1988). Discrete and continuous models of human information processing: Theoretical distinctions and empirical results. Acta Psychologica.

[CR15] Niehorster, D. C., Li, L., & Lappe, M. (2017). The accuracy and precision of position and orientation tracking in the HTC Vive Virtual Reality System for scientific research. *I-Perception*, *8*(3). https://doi.org/10.1177/2041669517708205 10.1177/2041669517708205 PMC5439658 28567271

[CR16] Oliva A, Wolfe JM, Arsenio HC (2004). Panoramic search: The interaction of memory and vision in search through a familiar scene. Journal of Experimental Psychology: Human Perception and Performance.

[CR17] O’Regan JK (1992). Solving the “real” mysteries of visual perception: The world as an outside memory. Canadian Journal of Psychology.

[CR18] Pausch, R., Snoddy, J., Taylor, R., Watson, S., & Haseltine, E. (1996). Disney’s Aladdin: First steps toward storytelling in virtual reality. In *Proceedings of the 23rd Annual Conference on Computer Graphics and Interactive Techniques*, 193–203. https://doi.org/10.1145/237170.237257

[CR19] Pellicano E, Smith AD, Cristino F, Hood BM, Briscoe J, Gilchrist ID (2011). Children with autism are neither systematic nor optimal foragers. Proceedings of the National Academy of Sciences of the USA.

[CR20] Shioiri S, Kobayashi M, Matsumiya K, Kuriki I (2018). Spatial representations of the viewer’s surroundings. Scientific Reports.

[CR21] Sidenmark L, Gellersen H (2020). Eye, head and torso coordination during gaze shifts in virtual reality. ACM Transactions on Computer-Human Interaction.

[CR22] Sisk CA, Remington RW, Jiang YV (2019). Mechanisms of contextual cueing: A tutorial review. Attention, Perception & Psychophysics..

[CR23] Sisk CA, Twedell EL, Koutstaal W, Cooper SE, Jiang YV (2018). Implicitly-learned spatial attention is unimpaired in patients with Parkinson’s disease. Neuropsychologia.

[CR24] Smith AD, Hood BM, Gilchrist ID (2008). Visual search and foraging compared in a large-scale search task. Cognitive Processing.

[CR25] Smith, A. D., Hood, B. M., & Gilchrist, I. D. (2010). Probabilistic cuing in large-scale environmental search. *Journal of Experimental Psychology. Learning, Memory, and Cognition*, *36*(3), 605–618. https://doi.org/10.1037/a0018280 10.1037/a0018280 20438260

[CR26] Treisman, A. (1988). Features and objects: The fourteenth Bartlett memorial lecture. *The Quarterly Journal of Experimental Psychology. A, Human Experimental Psychology*, *40*(2), 201–237. https://doi.org/10.1080/02724988843000104 10.1080/02724988843000104 3406448

[CR27] Vadillo MA, Linssen D, Orgaz C, Parsons S, Shanks DR (2020). Unconscious or underpowered? Probabilistic cuing of visual attention. Journal of Experimental Psychology: General.

[CR28] Wolfe, J., Cain, M., Ehinger, K., & Drew, T. (2015). Guided Search 5.0: Meeting the challenge of hybrid search and multiple-target foraging. *Journal of Vision*, *15*(12), 1106–1106. https://doi.org/10.1167/15.12.1106

[CR29] Won, B.-Y., & Jiang, Y. V. (2015). Spatial working memory interferes with explicit, but not probabilistic cuing of spatial attention. *Journal of Experimental Psychology. Learning, Memory, and Cognition*, *41*(3), 787–806. https://doi.org/10.1037/xlm0000040 10.1037/xlm0000040 PMC4420710 25401460

[CR30] Won B-Y, Lee HJ, Jiang YV (2015). Statistical learning modulates the direction of the first head movement in a large-scale search task. Attention, Perception & Psychophysics.

[CR31] Zang X, Shi Z, Müller HJ, Conci M (2017). Contextual cueing in 3D visual search depends on representations in planar-, not depth-defined space. Journal of Vision.

